# Persuasive Design Solutions for a Sustainable Workforce: Review of Persuasive Apps for Real-Time Capability Support for Rural Health Care Professionals

**DOI:** 10.2196/33413

**Published:** 2022-02-07

**Authors:** Sabrina Winona Pit, Aaron J H Tan, Robyn Ramsden, Kristy Payne, Winona Freihaut, Oliver Hayes, Benjamin Eames, Mike Edwards, Richard Colbran

**Affiliations:** 1 New South Wales Rural Doctors Network Hamilton Australia

**Keywords:** health, wellness, mobile apps, persuasive strategies, behavior change, review, health workforce, capability, career, employment, rural, workforce planning

## Abstract

**Background:**

There is a need to further investigate how persuasive design principles can change rural health professionals’ behaviors to look after their own health workforce capability. Several theories are used when developing apps to persuade people to change behavior, including the Persuasive System Design Model, consisting of primary task, dialogue, system credibility, and social support categories, and Cialdini’s principles of persuasion. These have not been analyzed yet in the field of health workforce capability.

**Objective:**

This study aims to determine the persuasive design techniques used in capability building–related apps and to provide recommendations for designing a health workforce app to increase their persuasiveness.

**Methods:**

A Python script was used to extract a total of 3060 apps from Google Play. Keywords centered around health workforce capability elements. App inclusion criteria were as follows: been updated since 2019, rated by users on average 4 and above, and more than 100,000 downloads. Next, 2 experts reviewed whether 32 persuasive strategies were used in the selected apps, and these were further analyzed by capability categories: competencies and skills, health and personal qualities, values and attitudes, and work organization.

**Results:**

In all, 53 mobile apps were systematically reviewed to identify the persuasive design techniques. The most common were surface credibility (n=48, 90.6%) and liking (n=48), followed by trustworthiness (n=43, 81.1%), reminders (n=38, 71.7%), and suggestion (n=30, 56.6%). The techniques in the social support domain were the least used across the different apps analyzed for health workforce capability, whereas those in the primary task support domain were used most frequently. The recommendations reflect learnings from our analysis. These findings provided insight into mobile app design principles relevant to apps used in improving health workforce capability.

**Conclusions:**

Our review showed that there are many persuasive design techniques that can assist in building health workforce capability. Additionally, several apps are available in the market that can assist in improving health workforce capability. There is, however, a specific lack of digital, real-time support to improve health workforce capability. Social support strategies through using social support persuasive design techniques will need to be integrated more prominently into a health workforce capability app. An app to measure and monitor health workforce capability scores can be used in conjunction with direct real-world person and real-time support to discuss and identify solutions to improve health workforce capability for rural and remote health professionals who are at high risk of burnout or leaving the rural health workforce.

## Introduction

Sustainability of the rural health workforce is paramount to meeting the health needs of rural Australia. Significant resources have been dedicated to identifying solutions to problems around recruitment or retention of the rural health workforce [1]. However, although extensive research has been conducted into the drivers behind retention and attrition of the rural health workforce, the literature does not adequately address how technology can be used to support rural health professionals in real time to improve their workforce capability. Health workforce capability can be defined as “the intersection between individual capacity and ability to respond to work, considering the whole of rural life, including work, family, schools, partner, education, and social options.” In other words, health workforce capability “describes a health professional’s overall level of capability in fulfilling their health care role.”

A 2021 scoping review found that digital solutions can enhance the capability and retention of rural health professionals [2]. The authors concluded that online platforms or digital solutions can address many of the challenges experienced by rural health professionals, by improving knowledge and skills, access, translation of knowledge into practice, empowerment, confidence, engagement, and provision of support. To this effect, for any digital app to be effective in improving rural health workforce capability, the desired behavior change that is needed must be considered during the development of the technical solutions. Many theories exist around user acceptance of IT solutions, such as the Mobile Application Rating Scale [3], the Technology Acceptance Model [4], and the Health Information Technology Acceptance Model [5]. However, they do not provide a clear systematic analysis and design criteria for developing persuasive software solutions to increase the likelihood of achieving the desired behavioral change [6].

One theory that can be used when developing apps to persuade people to change behavior include the Persuasive System Design Model (PSD-Model) [6]. This model can be used to identify the software functionality that may be useful in a product. Specifically, 28 design principles are provided and categorized into 4 main domains of persuasive techniques. These are the primary task, dialogue, system credibility, and social support domains. These 4 domains form part of the “design of system qualities” and have been applied to several health-related topics, such as arthritis [7] and mental health [8]. In their work on deconstructing persuasive principles and their implementation in health apps, Oyebode et al [9] expanded the scope of the PSD-Model by augmenting its 28 design principles with select techniques described in Cialdini’s principles of persuasion [10,11]. The authors selected only 4 persuasive techniques from Cialdini’s principles of persuasion because 2 were already present reflected in the PSD-Model, being authority and liking. This resulted in Oyebode et al [9] analyzing 32 persuasive design techniques in total. These 32 design techniques are classified into 5 domains, including the 4 from the PSD-Model listed above and Cialdini’s principles of persuasion. Specifically, Thach and Phan [8] reviewed the users’ perception of persuasiveness of mental health apps by qualitatively evaluating user reviews and concluded that when the principles of the PSD-Model are integrated into the design, users are happy with the design.

The question remains whether these design principles can be used when developing a technical solution to assist rural health professionals in maintaining or improving their health workforce capability. There are no clear data on this yet. A 2017 study that has provided some insight was a meta-analysis conducted by Carolan et al [12] of 21 randomized controlled trials (RCTs) involving web-based psychological interventions delivered in the workplace to improve employee psychological well-being and increase work effectiveness. In addition to exploring intervention effectiveness, the study identified features, including persuasive strategies, associated with greater engagement and adherence. The authors found that online interventions improve both psychological wellbeing and work effectiveness, but no differences were found between cognitive behavioral therapy versus other approaches, guidance versus no guidance, and targeted workplace populations versus the general workplace population. Work effectiveness was measured as work engagement, productivity, job-specific effectiveness, work-related self-efficacy, and work-related remuneration. Further analyses identified the following effective features that may likely increase engagement:

- Guidance delivered during a shorter period (6-7 weeks)
- Using secondary modalities to deliver the intervention (eg, emails, text messages, short messaging service)
- Elements of persuasive technology (eg, self-monitoring and tailoring)

Tailoring (57%), self-monitoring (43%), and tunneling (14%) were found to be used as persuasion strategies in studies included in the review and associated with the highest rates of engagement and adherence. Although the meta-analysis provided some useful insights, it did not focus on improving health workforce capability as such. There is a gap in the literature around how persuasive design techniques in digital solutions can change rural health professionals’ behaviors to look after their own health workforce capability.

Therefore, this study aimed to determine:

- The persuasive design techniques used to improve use of capability building–related apps; and
- Recommendations for incorporating persuasive design techniques in the design of health workforce capability apps.

## Methods

### Inclusion and Exclusion Criteria and Search Criteria

We based our methodology primarily on the study conducted by Oyebode et al [9]. A Python script was developed to extract apps from Google Play. Several rounds were conducted to refine the keywords (see Multimedia Appendix 1). Keywords centered around health workforce capability elements. The script limited searches to only English language, Australia only, and both free and paid apps. Apps with an average star rating of 4 and above were included to ensure only higher-quality apps from the user perspective were examined. Authors SP and RR reviewed 1091 apps to determine inclusion.

**Figure 1:**
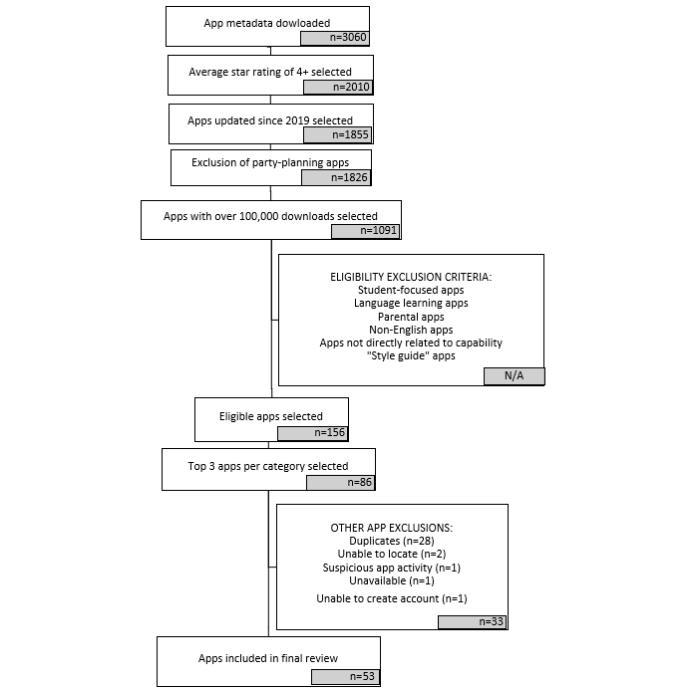
App selection and exclusion process.

Figure 1 describes the main steps in the app selection process, and Multimedia Appendix 1 provides additional information.

### Data Extraction, Coding, and Data Analyses of Selected Apps

Two theories were used in the apps to persuade people to change behavior: the PSD-Model [6] and Cialdini’s principles of persuasion [11]. Both theories have been extensively used when analyzing persuasive technologies [9]. Oyebode et al [9] determined that 32 techniques can be measured based on both theories, as the techniques authority and liking were common to both. Next, 2 authors (WF and OH) with a data sciences and psychology background reviewed whether the 32 persuasive design techniques were applied in the apps.

The reviewers downloaded the apps on an Android smartphone or emulator and used the apps to determine whether the persuasive design techniques were present in the apps. The reviewers coded independently and then came together to compare scores to ensure interrater reliability. If there was a discrepancy, a discussion was held to reach consensus. The coders double-coded all apps and achieved a κ of .81, indicating very good agreement between the 2 coders.

### Data Analyses

After scoring the 53 apps, the apps were divided in 4 broad workforce capability app categories to determine whether there were differences between the different persuasive design techniques used within each category, including competencies and skills, health and personal qualities, values and attitudes, and work organization. This classification was chosen as it aligned with findings from a qualitative data analysis that had been simultaneously conducted to explore user perspectives on building a health workforce capability app (Ramsden et al, unpublished data, 2021). The classification also mirrors elements of the work ability to explain sustainable employability among general practitioners [13]. Competencies and skills include elements such as clinical competence, drug and clinical references, and medical journals. Health and personal quality apps are related to the health professionals’ own lives and not patient-related or medical knowledge–type apps. Work organization includes elements such as patient-centered care and entrepreneurial skills. Value and attitude apps include elements such as resilience and self-motivation.

Following this classification, data were analyzed as follows:

- Total number of persuasive design techniques used per domain: primary task support, dialogue support, system credibility support, social support, and Cialdini’s principles of persuasion
- Total number and percentage of each persuasive design technique used in each health workforce capability category: competence and skill, health and personal qualities, values and attitudes, and work organization
- Total number of persuasive design techniques used by the persuasive design technique domain across the 4 workforce capability categories developed for this systematic review: competencies and skills, health and personal qualities, values and attitudes, and work organization

Following the quantitative analyses of apps, implementation examples of the PSD-Model, consisting of primary task, dialogue, system credibility, and social support categories, and Cialdini’s principles of persuasion are provided.

### Ethics

Ethical approval to conduct the feasibility study was received from the Northern NSW Local Health District Human Research Ethics Committee (2020/ETH03020).

## Results

### Overall Results

**Figure 2:**
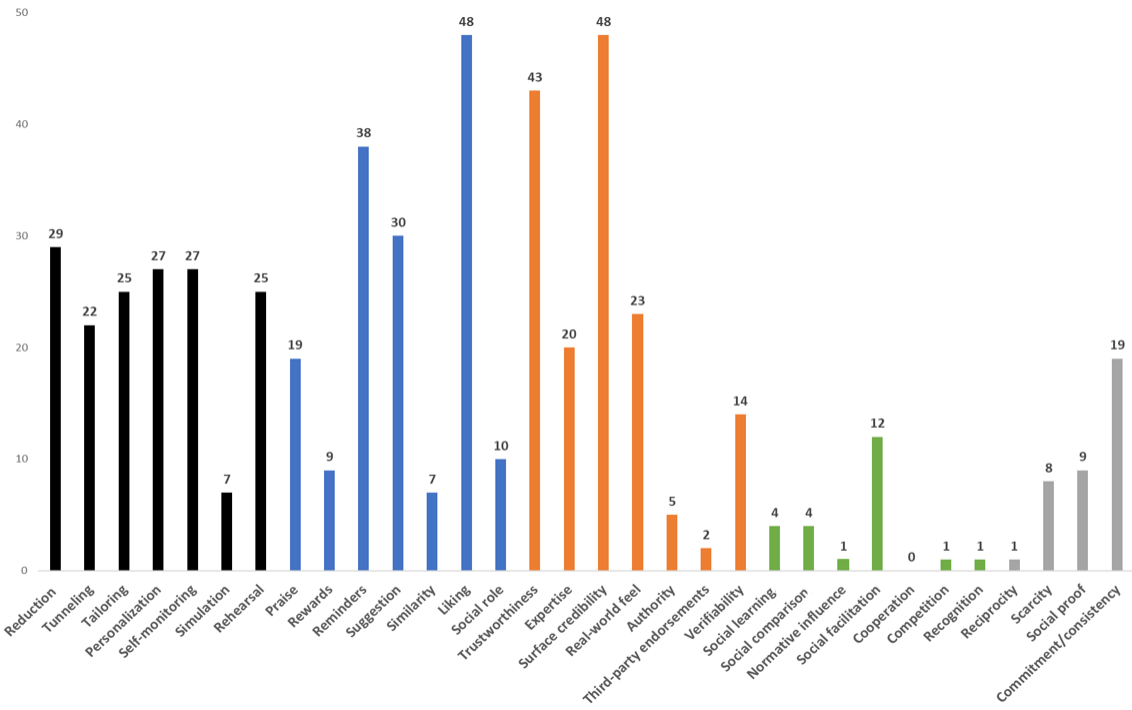
Total number of persuasive design techniques used by category.

Figure 2 shows that the most common persuasive design techniques were surface credibility (48/53, 90.6%) and liking (48/53, 90.6%), followed by trustworthiness (43/53, 81.1%), reminders (38/53, 71.7%), and suggestion (30/53, 56.6%). Social support persuasive design techniques were the least used across the different apps analyzed for health workforce capability, whereas primary task support design techniques were used most frequently overall, with the exception of simulation.

Table 1 shows the most used persuasive techniques per workforce capability category. Those that were used in 50% or more of the total 53 apps are formatted in italic. It is evident that health apps included the most persuasive techniques, as did the apps related to values and attitudes. Apps related to work organization included the least number of design techniques.

**Table 1:** Persuasive design techniques used by the health workforce capability group.

Persuasive design technique		Count, n (%)
**Competence and skill (n=16)**		
	*Surface credibility* ^a^	16 (100)
	*Liking*	15 (94)
	*Trustworthiness*	14 (88)
	*Reminders*	10 (63)
	*Tailoring*	9 (56)
	*Personalization*	9 (56)
	*Tunneling*	8 (50)
	*Expertise*	8 (50)
	Reduction	7 (44)
	Rehearsal	7 (44)
	Suggestion	7 (44)
	Self-monitoring	6 (38)
	Real-world feel	6 (38)
	Verifiability	6 (38)
	Simulation	4 (25)
	Praise	4 (25)
	Scarcity	4(25)
	Rewards	3 (19)
	Authority	3 (19)
	Commitment/consistency	3 (19)
	Similarity	2 (13)
	Social role	2 (13)
	Social proof	2 (13)
	Social facilitation	1 (6)
	Third-party endorsements	0 (0)
	Social learning	0 (0)
	Social comparison	0 (0)
	Normative influence	0 (0)
	Cooperation	0 (0)
	Competition	0 (0)
	Recognition	0 (0)
	Reciprocity	0 (0)
**Health and personal qualities (n=17)**		
	*Liking*	17 (100)
	*Surface credibility*	17 (100)
	*Reminders*	15 (88)
	*Trustworthiness*	15 (88)
	*Suggestion*	13 (76)
	*Reduction*	12 (71)
	*Self-monitoring*	11 (65)
	*Personalization*	10 (59)
	*Real-world feel*	10 (59)
	*Rehearsal*	9 (53)
	*Commitment/consistency*	9 (53)
	Tunneling	8 (47)
	Tailoring	8 (47)
	Praise	7 (41)
	Expertise	5 (29)
	Social facilitation	5 (29)
	Social proof	4 (24)
	Social role	3 (18)
	Verifiability	3 (18)
	Social learning	3 (18)
	Scarcity	3 (18)
	Rewards	2 (12)
	Social comparison	2 (12)
	Similarity	1 (6)
	Authority	1 (6)
	Reciprocity	1 (6)
	Simulation	0 (0)
	Third-party endorsements	0 (0)
	Normative influence	0 (0)
	Cooperation	0 (0)
	Competition	0 (0)
	Recognition	0 (0)
**Values and attitudes (n=10)**		
	*Liking*	10 (100)
	*Surface credibility*	8 (80)
	*Self-monitoring*	7 (70)
	*Reminders*	7 (70)
	*Trustworthiness*	7 (70)
	*Reduction*	6 (60)
	*Tailoring*	6 (60)
	*Rehearsal*	6 (60)
	*Praise*	5 (50)
	*Suggestion*	5 (50)
	*Commitment/consistency*	5 (50)
	Tunneling	4 (40)
	Personalization	4 (40)
	Real-world feel	4 (40)
	Rewards	3 (30)
	Social role	3 (30)
	Expertise	3 (30)
	Verifiability	3 (30)
	Similarity	2 (20)
	Social facilitation	2 (20)
	Simulation	1 (10)
	Third-party endorsement	1 (10)
	Social comparison	1 (10)
	Social proof	1 (10)
	Authority	0 (0)
	Social learning	0 (0)
	Normative influence	0 (0)
	Cooperation	0 (0)
	Competition	0 (0)
	Recognition	0 (0)
	Reciprocity	0 (0)
	Scarcity	0 (0)
**Work organization (n=10)**		
	*Trustworthiness*	7 (70)
	*Surface credibility*	7 (70)
	*Reminders*	6 (70)
	*Liking*	6 (60)
	*Suggestion*	5 (50)
	Reduction	4 (40)
	Personalization	4 (40)
	Expertise	4 (40)
	Social facilitation	4 (40)
	Rehearsal	3 (30)
	Self-monitoring	3 (30)
	Praise	3 (30)
	Real-world feel	3 (30)
	Tunneling	2 (20)
	Tailoring	2 (20)
	Simulation	2 (20)
	Similarity	2 (20)
	Social role	2 (20)
	Verifiability	2 (20)
	Social proof	2 (20)
	Commitment/consistency	2 (20)
	Rewards	1 (10)
	Authority	1 (10)
	Third-party endorsement	1 (10)
	Social learning	1 (10)
	Social comparison	1 (10)
	Normative influence	1 (10)
	Competition	1 (10)
	Recognition	1 (10)
	Scarcity	1 (10)
	Cooperation	0 (0)
	Reciprocity	0 (0)

^a^Design techniques used in 50% or more of the total 53 apps are formatted in italic.

### Persuasive Design Techniques’ Domains by Workforce Capability Category

**Figure 3:**
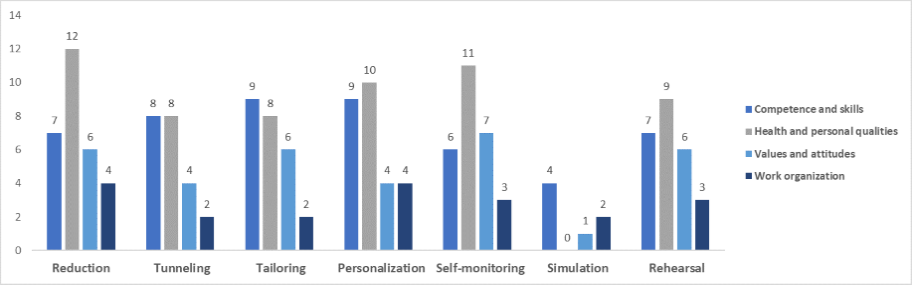
Total number of persuasive design techniques used by the primary task support category.

**Figure 4:**
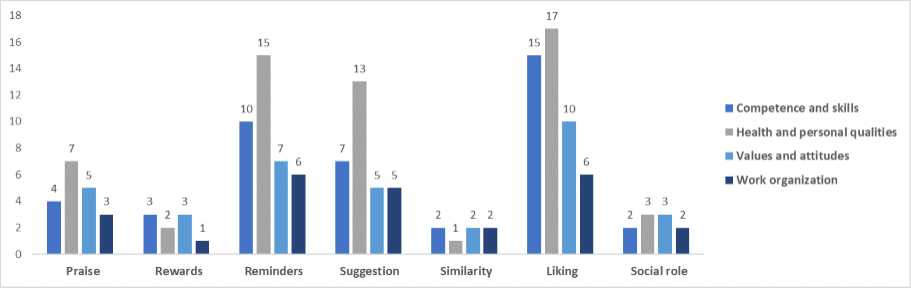
Total number of persuasive design techniques used by the dialogue support category.

**Figure 5:**
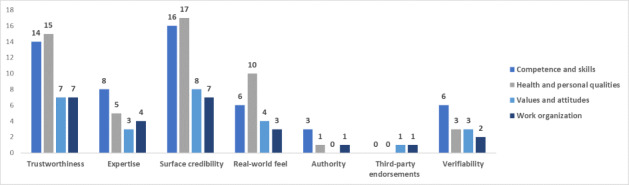
Total number of persuasive design techniques used by the system credibility support category.

**Figure 6:**
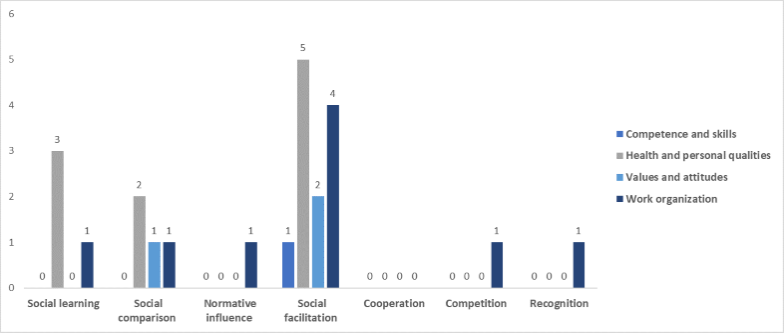
Total number of persuasive design techniques used by the of social support category.

**Figure 7:**
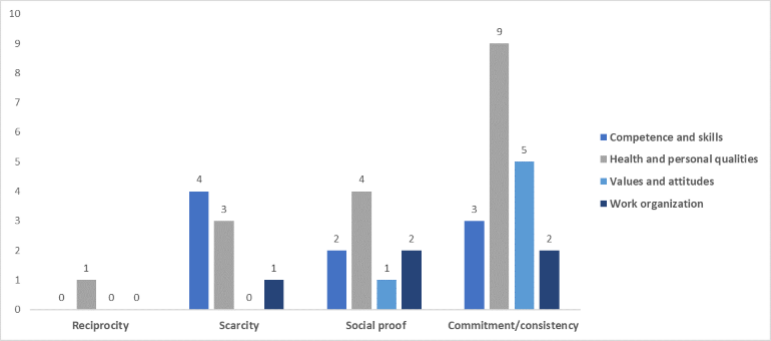
Total number of persuasive design techniques used by Cialdini’s principles of persuasion.

Figures 3-7 show the various persuasive design techniques’ domains across the 4 workforce capability categories developed for this systematic review: competencies and skills, health and personal qualities, values and attitudes, and work organization.

The following observations were made:

- Primary task support techniques demonstrated use among all workforce capability categories, with *reduction* and *self-monitoring* being less evident in competence-related apps when compared to the health apps.
- For dialogue support, the most frequently used technique was liking, which was high among all categories followed by *reminders* (Figure 5).
- Among system credibility support techniques, *surface credibility* featured most strongly among health (n=16) and competence (n=16) apps, which was similar for *trustworthiness* (n=15 for health and n=14 for competences), while *real-world feel* was most frequently observed in health apps (Figure 6).
- Social support techniques did not feature strongly across all domains, with the exception of *social facilitation* (n=5 for health and n=4 for work organization) (Figure 7).
- Among Cialdini’s principles of persuasion, *commitment and consistency* played a large role in health and personal quality–related apps (n=9), followed by value- and attitude-related apps (n=5), while *reciprocity* barely played a role in any workforce capability category (Figure 7).

### Persuasive Design Techniques and Implementation Suggestions Based on Existing Apps

Tables 2-6 show the various persuasive design techniques used in the apps analyzed in this study and how these can be extrapolated to create suggestions for developing a health workforce capability app.

**Table 2:** Persuasive design techniques: definitions and implementation examples for developing a health workforce capability app in the primary task support domain.

Persuasive design technique	Persuasive design technique definition^a^	Implementation examples
Reduction	A system that reduces complex behavior into simple tasks helps users perform the target behavior, and it may increase the benefit/cost ratio of a behavior.	Health workforce capability goals are broken down into smaller steps.
Tunneling	Using the system to guide users through a process or experience provides opportunities to persuade along the way.	A user should be able to choose a pathway that would respond to their specific health workforce capability need.
Tailoring	Information provided by the system will be more persuasive if it is tailored to the potential feeds, interests, personality, usage context, or other factors relevant to a user group.	A user should be able to choose what capability area they are particularly interested in.
Personalization	A system that offers personalized content or services has a greater capability for persuasion.	The system adjusts to the health care profession and the user’s age and offers localized services to improve health workforce capability.
Self-monitoring	A system that keeps track of one’s own performance or status supports the user in achieving goals.	The system asks health professional to rate their health workforce capability.
Simulation	Systems that provide simulations can persuade by enabling users to observe immediately the link between cause and effect.	The system allows for health professionals to observe other health professionals working interprofessionally and see improved patient outcomes.
Rehearsal	A system providing means with which to rehearse a behavior that can enable people to change their attitudes or behavior in the real world.	A telehealth simulation course is offered to rehearse real-world practice and improve use of telehealth.

^a^Source: Oinas-Kukkonen and Harjumaa [6].

**Table 3:** Persuasive design techniques: definitions and implementation examples for developing a health workforce capability app in the dialogue support domain.

Persuasive design technique	Persuasive design technique definition^a^	Implementation examples
Praise	By offering praise, a system can make users more open to persuasion.	Texts and symbols are used to offer praise after measuring their own capability score.
Rewards	Systems that reward target behaviors may have great persuasive powers.	The game rewards users by altering media items, such as sounds and background colors.
Reminders	If a system reminds users of their target behavior, the users will more likely achieve their goals.	The user is given a reminder of a selected task to improve capability.
Suggestion	Systems offering fitting suggestions will have greater persuasive powers.	Suggestions are given to be mindful at work or build capability.
Similarity	People are more readily persuaded through systems that remind them of themselves in some meaningful way.	Videos/pictures of health professionals are shown.
Liking	A system that is visually attractive for its users is likely to be more persuasive.	The application has an integrated system that links well with easy-to-read graphs and trends in health workforce capability.
Social role	If a system adopts a social role, users will more likely use it for persuasive purposes.	A dementia expert supports online education for trainees with dementia.

^a^Source: Oinas-Kukkonen and Harjumaa [6].

**Table 4:** Persuasive design techniques: definitions and implementation examples for developing a health workforce capability app in the system credibility support domain.

Persuasive design technique	Persuasive design technique definition^a^	Implementation examples
Trustworthiness	A system that is viewed as trustworthy will have increased powers of persuasion.	The system includes a privacy statement. The app demonstrates that the organization has access to funding to support health professionals in their health workforce capability and demonstrates successful examples.
Expertise	A system that is viewed as incorporating expertise will have increased powers of persuasion.	The app demonstrates that the organization has a longstanding reputation in providing health workforce capability support.
Surface credibility	People make initial assessments of the system credibility based on a firsthand inspection.	There are no commercial ads.
Real-world feel	A system that highlights people or the organization behind its content or services will have more credibility.	Users are able to contact the organization to request health workforce capability support.
Authority	A system that leverages roles of authority will have enhanced powers of persuasion.	A health professional national college provides a statement on the importance of health workforce capability.
Third-party endorsements	Third-party endorsements, especially from well-known and respected sources, boost perceptions on system credibility.	A well-respected, known, experienced rural health professional endorses the app.
Verifiability	Credibility perceptions will be enhanced if a system makes it easy to verify the accuracy of site content via outside sources.	The app offers links and support by well-established services.

^a^Source: Oinas-Kukkonen and Harjumaa [6].

**Table 5:** Persuasive design techniques: definitions and implementation examples for developing a health workforce capability app in the social support domain.

Persuasive design technique	Persuasive design technique definition^a^	Implementation examples
Social learning	A person will be more motivated to perform a target behavior if they can use a system to observe others performing the behavior.	The app includes stories of other rural health professionals who have improved their workforce capability.
Social comparison	System users will have a greater motivation to perform the target behavior if they can compare their performance with the performance of others.	Users can share information real time on how to do something more efficient.
Normative influence	A system can leverage normative influence or peer pressure to increase the likelihood that a person will adopt a target behavior.	The app shows that self-care is key to long-term employability by using examples of other professionals.
Social facilitation	System users are more likely to perform a target behavior if they discern via the system that others are performing the behavior along with them.	Health professionals know that many other people are also participating in the app and can choose to discuss with other users.
Cooperation	A system can motivate users to adopt a target attitude or behavior by leveraging human beings’ natural drive to cooperate.	The app demonstrates that working in a team leads to better patient health outcomes.
Competition	A system can motivate users to adopt a target attitude or behavior by leveraging human beings’ natural drive to compete.	Health workforce capability scores can be used to determine the personal best in a specific area they wish to work on.
Recognition	By offering public recognition for an individual or group, a system can increase the likelihood that a person/group will adopt a target behavior.	The app demonstrates the “Rural health professional of the month” and how they benefited from improving their health workforce capability.

^a^Source: Oinas-Kukkonen and Harjumaa [6].

**Table 6:** Persuasive design techniques: definitions and implementation examples for developing a health workforce capability app in the domain of Cialdini’s principles of persuasion.

Persuasive design technique	Persuasive design technique definition^a^	Implementation examples
Commitment/consistency	These are a pair of interrelated attributes in the sense that people often adhere (consistently) to their significant choices (commitments).	The app has the ability to record health workforce capability goals (commitment) and timings (consistency) so that health professionals can commit to goals.
Scarcity	This causes people to almost panic out of the fear that something will disappear or become unavailable, so they make an intent effort to acquire or preserve it.	—^b^
Social proof	This explains the human tendency to look around at others in society for reinforcement and direction in taking action.	The app shows the number of health professionals that have joined the health workforce capability app.
Reciprocity	This describes the human desire to make others feel appreciated by responding in ways that return good gestures.	The app allows users to post their own health workforce capability issues and also to respond to other users’ posts.

^a^Source: Oyebode et al [9].

^b^Not available.

A wide variety of implementation examples were drawn from analyzing the apps. In summary, the *primary task support* domain focuses on techniques that help the health professional focus on an element of health workforce capability that is important to them. The *dialogue support* techniques are about the dialogue that occurs between the health professional and the digital system to improve the health professional’s ability to work on their health workforce capability. *System credibility support* techniques include using only high-quality products and services and stakeholders that are associated with the app, for example, respected health professionals, health organizations, and Australian clinical guidelines endorsed by colleges representing clinical groups. *Social support* techniques play a role in showcasing how and how many other health professionals manage their health workforce capability and facilitating collaboration and information sharing between health professionals.

## Discussion

### Principal Findings

A systematic review of 53 apps that are related to health workforce capability was performed through deconstruction of persuasive design techniques and their implementation. The findings demonstrated that health professional needs and digital solutions broadly align with the 4 health workforce capability categories used in this study: competencies and skills, health and personal qualities, values and attitudes, and work organization. These categories were matched with 32 persuasive design techniques and provided suggestions on how to further improve the persuasiveness of apps used to improve health workforce capability.

#### Persuasive Design Techniques

Of the 53 apps, the most common persuasive design techniques were surface credibility (n=48, 90.6%) and liking (n=48), followed by trustworthiness (n=43, 81.1%), reminders (n=38, 71.7%), and suggestion (n=30, 56.6%). Social support persuasive design techniques were the least used across the different apps analyzed for health workforce capability, whereas primary task support techniques were the most common, with the exception of simulation. The apparent lower inclusion of persuasive design techniques around social support is a contrast, given that previous qualitative analyses have shown that rural health professionals perceive social and professional isolation to play a major role in building and maintaining their health workforce capability (Ramsden et al, unpublished data, 2021), and also provide multiple suggestions on how this could be achieved in this study. Examples given were related to online communities of practices, interprofessional learning (social comparison and learning), and telehealth improving trust between different disciplines, but also around having an online career coach (normative influence) and demonstrating online effectiveness of team performance and peer recognition, for example, by showcasing good news stories in health workforce capability. This study was based on the analyses by Oyebode et al [9], who reviewed 80 popular mHealth apps using the 32 techniques as described above. Briefly, the most common persuasive categories identified in Oyebode et al’s [9] study were personalization (n=77, 96.3%), surface credibility (n=69, 86.3%), trustworthiness (n=66, 82.5%), self-monitoring (n=64, 80%), real-world feel (n=59, 73.8%), reminders (n=57, 71.3%), suggestion (n=56, 70%), liking (n=52, 65%), expertise (n= 52), commitment/consistency (n=47, 58.8%), reduction (n=45, 56.3%), and tunneling (n=40, 50%). The authors provided persuasive strategy suggestions that can be used by app developers to increase the likelihood of behavior change. Similar to our study, Oyebode et al [9] pointed out that social interaction can motivate people to reach their behavioral targets, yet social support is rarely used in mHealth apps (17 of 80 apps, 21%). They thus recommend for future apps to include social support strategies.

Across health workforce capability domains, we found that health apps include the most persuasive techniques, as do the apps related to values and attitudes. Interestingly, the apps related to work organization have the least persuasive techniques, even though work organization plays a large role in maintaining or building health workforce capability [13]. For example, the ability to link to local allied health professionals through an online booking system would enhance a general practitioner’s (GP) workforce capability (Ramsden et al, unpublished data, 2021) by improving interdisciplinary, holistic care. The online system would also facilitate sending and sharing reports between the GP and an allied health professional. The online booking system would also reduce the likelihood of the patient not following up with an allied health professional as they get an appointment on the spot (social facilitation persuasive design technique between the GP, allied health professional, and patient). When designing digital tools that focus on work organization to increase capability, designers could focus on including persuasive design techniques, such as real-world feel, tunneling, and tailoring, which are currently underrepresented in the work organization category.

It appears that health apps include a wide array of persuasive techniques, and thus, building a health workforce capability app could learn from the design of capability-related health aps. It should be kept in mind, however, that Oyebode et al [9] did not find a direct relationship between the number of persuasive strategies used and app effectiveness (measured by user app ratings). The authors, therefore, argued that adopting fewer persuasive design techniques could potentially be as effective, as it potentially reduces the complexity of the app and thus prevents user cognitive overload. The number of techniques used is still under debate, and more research is needed in this area.

#### Health Workforce Capability Digital Support Tool

Khakurel et al [14] conducted a systematic review of 34 wearable device studies and concluded that wearable technology can potentially increase work efficiency for staff, improve workers’ physical well-being, and reduce work-related injuries. However, they also reported that technological, social, policy, data, and economic issues related to wearable technology remain. The authors developed a categorization of wearable devices, including monitoring, assisting, augmenting, tracking, and delivering content. Alhasani et al [15] applied a similar systematic review to 60 stress apps. The authors found that most apps use manual tracking, and pointed out that this can become boring and users might forget to log data. A combination of manual entry and automated tracking sensors (hybrid data capture) is recommended to improve the likelihood of reaching a set behavioral target change. For example, accelerometer data in a mobile phone can be used to predict the stress level at work [16] through the use of a data collection app stored on the phone that measures the continuous process of recording active apps, starting and ending time stamps, and duration of app use. By linking these data with the participant’s self-reported stress levels, researchers could accurately predict future stress levels. Ferdous et al [16] demonstrated an average accuracy of 75% and a precision of 85.7% as indicators of overall stress levels in work environments. This information can be used to inform stress reduction, organizational policies, and the interrelation between stress and productivity of workers. Reactions from health professionals varied according to their willingness to trial wearables, but at the same time, qualitative analyses identified that health professionals are receptive to certain elements of persuasive design techniques that relate to wearables, such as monitoring, feedback, reminders, reduction, and tunneling.

Content filtering and personalized interventions that match users’ needs have been demonstrated to improve persuasion power [15], such as the persuasive design techniques analyzed in our study: tailoring, tunneling, and reduction. The authors also recommend that users can customize the user interface and app features, such as color background, or their user profile as this gives them increased self-agency, a sense of control, and identity. Persuasive design techniques such as these could be implemented within a capability-building digital solution.

We propose to use a strengths-based approach to improving rural health professionals’ capability and include persuasive design techniques in digital solutions. For example, future applications can measure the health professional’s vitality or health workforce capability scores at different times throughout the day by asking them to rate their capability at random times or a set time throughout the week. Alternatively, some health professionals may be willing to use a hybrid model by augmenting self-ratings with wearable technology, such as vital signs or step counts. In either case, the health professional can view graphs or trends and analyses showing self-rated health workforce capability levels over time and time of day and display simple statistics, such as minimum, maximum, and average scores. This would reflect the persuasive design technique monitoring. Moving forward, the data can potentially also be used to predict future health workforce capability and recommend specific behavioral change interventions for the health professional to allow for tunneling and reduction. Furthermore, if the health workforce capability score falls, a reminder could pop up for the health professional to take appropriate action. This could include contacting a trusted organization or coach (system credibility persuasive techniques, such as trustworthiness and real-world feel, as linkage occurs with real people) to discuss the health workforce capabilities that they feel are hindering their current capability and identify solutions to increase their capability. It could also involve the health professional doing some exercise or other activity that helps them with their well-being and feelings of capability. Other simple persuasive design techniques, such as limited ads, will increase system credibility support through increased surface credibility. Given that health workforce capability is determined by the interplay of a complex array of factors [2] (Ramsden et al, unpublished data, 2021), this may well be the first step toward building a futuristic, completely digitalized tool to improve the health workforce capability support tool.

#### Ethical Considerations

There are many ethical aspects that needs to be considered in developing a digital solution supported by real-time support by real people [17].

First, further research is needed on the ethical aspects of this type of data capture, as this will have major security implications. Indeed, Alhasani et al [15] note that every interaction with the app creates behavioral data, via audit trails or sensors, that can be analyzed in real time to predict users’ needs. This means that sensitive information about a user is being generated and stored.

Second, there is likely to be a perceived conflict of interest if an organization would own a health workforce capability digital tool and also provide the support to help health professionals with their capability.

Third, the active participation of a health profession can be strongly affected by factors such as the health professionals’ perception of how the data are owned, stored, and used. There is thus a need to balance the individual privacy of the data generated with the benefits that an amalgamated data set could provide to the workforce as a whole. As discussed, the ethics of using such granular data need to be further evaluated, and an acceptability study regarding the scope of use for the collected data would be timely. Nonetheless, as alluded to before, it is key to ensure that the data are kept secure and only used for their intended purpose, whatever that is determined to be. Users must also be reassured of this and their confidence maintained.

Lastly, relying on self-rated measures may reinforce unconscious incompetence [18] or result in social desirable answers where a health professional knowingly scores themselves optimistically due to the perception of negative effects on their professional registration or professional standing. This can be mitigated by building a culture of trust and support between the organization and the health professional and a shared understanding that these analyses are being done for the benefit of the health professional.

### Limitations

Although some may see that using only apps from Google Play Store may lead to potential bias in the analyses by excluding Apple App Store products, Meacham et al [19] report that many popular apps can be found on both platforms. They report that developers perceive that it is easier to register their product with Google Play Store than with the Apple App Store. This in turn may lead to some viewing the Apple App Store as of better quality and more unlikely to be free [19]. To ensure the analyses only included higher-quality apps as perceived by the end user, our review excluded apps with user star ratings below 4.

The apps were selected based on search strategy, number of downloads, and reviewer ratings and could be viewed as a proxy for quality. We did not perform a quality assessment of the apps, as this was outside the scope of this review, nor do we endorse any of the apps analyzed. The intent of this review was to analyze the persuasive strategies used in apps that contribute to health workforce capability. Although this can be seen as a study limitation, health workforce capability is a broad and complex concept and this app review should be viewed as exploratory. Furthermore, although the script to select apps from Google Play for the review focused on selecting apps that are available in Australia, the results are transferable to other countries from a persuasive design technique viewpoint. Countries will have their own (clinical) guidelines that may be important for health workforce capability.

It should be considered that the numbers of apps analyzed was relatively small and is therefore only an exploratory investigation of the persuasive design principles by the health workforce capability domain. Nonetheless, the strength lies in the combination of looking at the persuasive design techniques used and in relation to their health workforce capability needs.

A further limitation of the study is that it was beyond the scope to draw conclusions about the impact the number of persuasive techniques included has on app effectiveness. Further research is needed to explore this in relation to health workforce capability apps.

### Conclusion

There are many persuasive design techniques that can assist in building capability. Commonly used techniques are surface credibility, liking, trustworthiness, reminders, and suggestion, while less common are social support persuasive design techniques. Additionally, several apps are available in the market that can assist in improving health workforce capability. There is, however, a specific lack of digital, real-time support to improve health workforce capability. Social support strategies through using social support persuasive design techniques will need to be integrated more prominently into a health workforce capability app. An application to measure and monitor health workforce capability scores can be used in conjunction with direct real-world person and real-time support to discuss and identify solutions to improve health workforce capability for rural and remote health professionals who are at high risk of burnout or leaving the rural health workforce.

## Appendix

Multimedia Appendix 1 Selection process.

## Abbreviations

GP: general practitioner
PSD-Model: Persuasive System Design Model
RCT: randomized controlled trial
