# Supplementary material for: Persuasive Design Solutions for a Sustainable Workforce: Review of Persuasive Apps for Real-Time Capability Support for Rural Health Care Professionals
Source: JMIR Mhealth Uhealth. 2022 Feb 7;10(2):e33413. doi: 10.2196/33413 (PMC8861870; doi:10.2196/33413)
Supplement: Multimedia Appendix 1 [file mhealth_v10i2e33413_app1.docx]

# **Appendix A**

The python developer used the keywords in Google App Store and had to limit the search to 60 searches per term as Google 503 banned the IP a few times.

**Table 1. App Categories and Search keywords**

| **App Category** | **Description Google Play** | **Search terms** |
| --- | --- | --- |
| Health and Fitness | Personal fitness, workout tracking, diet and nutritional tips, health & safety, etc. | Resilient, resilience, self-motivation, self-care, wellness, physical health, healthy diet, healthy nutrition, healthy sleep, healthy mental health, relaxation for health, meditation for health and mindfulness for health. |
| Medical | Drug & clinical references, calculators, handbooks for health-care providers, medical journals & news | Clinical competence, patient centred care and medical ethics. |
| Lifestyle | Style guides, wedding & party planning, how-to guides | Personal vision alignment, decision making skills, confidence, teamwork skills, relationship skills, team influencing skills, positive relationships, and positive attitude. |
| Education | Exam preparations, study-aids, vocabulary, educational games, language learning | Workplace wellness, work life balance, teamwork skills, team communication skills, entrepreneurial skills, innovation skills, communication skills, listening skills and emotional intelligence. |

Figure 1 in the main text describes the app selection process. Briefly,

1. 3010 app were downloaded on 5 May 2021. Inclusion criteria were:
   1. Star-rating 4 and above to ensure there were enough apps in all domains that relate to health workforce capability, otherwise, for example, only health and fitness apps would have been left
   2. Updated since 2019
2. The following search categories were combined:
   1. healthy diet, healthy nutrition and nutrition and diet
   2. Healthy mental health and mental health
   3. Mindfulness for health and mindfulness
   4. Resilience and resilient
   5. Personal fitness, workout tracking and physical fitness
   6. Positive attitude and positive relationships
   7. Team communication skills, team influencing skills and teamwork skills
   8. Relaxation for health and wellness
3. There were selected groups of apps that had a medical classification category: including clinical competence, drug and clinical references, handbooks for health care providers, medical calculators, medical ethics, medical journals, medication for health and patient centred care.
4. Apps with less than 100,000 downloads were excluded. This left 1091 apps for further analyses.
5. For each category, apps were then listed in order of number of downloads followed by reviewers’ score.
6. For each search term, maximum three apps were included, allowing for a cross section of health workforce capability terms. However, for work-life balance more apps were included as they reflected an important component of the health workforce capability concept
7. Two authors (SP, RR) went through the 1091 apps and their titles to decide eligibility (see Figure 1) for further investigation. If it was unclear, the website was consulted. When five apps were reached per category, the authors moved to the next category.
8. This led to 156 apps across different categories. Of these the top three were selected per category for further analyses which included 86 apps. Another 33 were excluded mainly because they were duplicates, leaving 53 apps for further analyses.
